# Glucagon-like peptide-1 receptor agonists and the risk of erectile dysfunction: a drug target Mendelian randomization study

**DOI:** 10.3389/fendo.2024.1448394

**Published:** 2024-11-13

**Authors:** Hongjin An, Kexin Xie, Huatian Gan

**Affiliations:** ^1^ Department of Gastroenterology and Hepatology, West China Hospital, Sichuan University, Chengdu, China; ^2^ Department of Geriatrics and National Clinical Research Center for Geriatrics, West China Hospital, Sichuan University, Chengdu, China; ^3^ Department of Gastroenterology and Laboratory of Inflammatory Bowel Disease, the Center for Inflammatory Bowel Disease, Clinical Institute of Inflammation and Immunology, Frontiers Science Center for Disease-related Molecular Network, West China Hospital, Sichuan University, Chengdu, China

**Keywords:** glucagon-like peptide-1 receptor agonists, erectile dysfunction, type 2 diabetes, obesity, Mendelian randomization

## Abstract

**Background:**

Glucagon-like peptide-1 receptor agonists (GLP-1RAs) have been widely used for type 2 diabetes (T2D) and weight management. However, the causal relationship of GLP-1RAs with erectile dysfunction (ED) was still unclear.

**Methods:**

Mendelian randomization (MR) analysis was conducted to reveal the association of genetically proxied GLP-1RAs with ED. The proportion of potential mediators mediating GLP-1RAs to ED was also assessed by two-step MR. Finally, a series of sensitivity analyses and Two-Sep cis-MR (TSCMR) were performed to evaluate the robustness of the results.

**Results:**

MR evidence suggested that genetically proxied GLP-1RAs reduced the risk of ED [odds ratio (OR): 0.493; 95% confidence interval (95% CI): 0.430 to 0.565; *P*<0.001]. Further mediation analysis via two-step MR showed that this effect was partly mediated through reduced T2D, obesity, hypertension and cardiovascular disease (CVD), with mediated proportions of 2.89% (95% CI: 1.28% to 4.49%), 6.83% (95% CI: 2.25% to 11.41%), 3.22% (95% CI: 1.21% to 5.23%), and 3.06% (95% CI: 0.51% to 5.62%), respectively.

**Conclusions:**

GLP-1RAs were associated with a reduced risk of ED, and to a lesser extent, T2D, obesity, hypertension and CVD mediated this effect. Nevertheless, the potential implications of our results for ED prevention policies required validation in further clinical randomized controlled trials.

## 1 Introduction

Erectile dysfunction (ED), defined as the inability to achieve and maintain an erection sufficient for sexual intercourse, is estimated to occur in 20% of men over the age of 40 and the incidence increases with age (1, 2). This rate is even higher among patients with type 2 diabetes (T2D) and obesity. In fact, more than 50% of men with T2D and obesity complain of ED (3, 4). Currently, the use of glucagon-like peptide-1 receptor agonists (GLP-1RAs) has increased dramatically due to their potent glucose-lowering and weight-loss effects, especially in young groups used for weight management (5). However, there is a paucity of research evidence on whether GLP-1RAs are friends or foes to ED. This inevitably raises concerns about the use of GLP-1RAs in populations with a high incidence of ED.

A recent retrospective cohort study suggested that GLP-1RAs might have induced positive vasculature effects, thereby improving erectile function in patients with T2D (6). However, due to the limitations of the retrospective study itself, the small sample included (108 outpatients) and the lack of a third group of patients on GLP-1RAs only, the study was very narrow in its interpretation of the relationship between GLP-1RAs and ED and could not make causal inference (6). Although another exploratory analysis showed that long-term use of dulaglutide (belongs to GLP-1RAs) might reduce the incidence of ED in men with T2D (7), given the presence of a history of cardiovascular events as well as the use of β blockers in the samples enrolled in this study, the assessment of the impact of GLP-1RAs on ED remained very limited and could not be generalized to individuals who only used GLP-1RAs for weight loss but did not have cardiovascular disease or use of cardiovascular related drugs. Furthermore, considering the potential adverse microvascular effects of GLP-1RAs (8) and the paucity of experimental evidence on autonomic neuropathy (9), the effect of GLP-1RAs on ED remains uncertain (9). Therefore, it is necessary to further explore the effects of GLP-1RAs on ED, which is critical to the safety regulation of the dramatic increase in the frequency of GLP-1RAs use.

Mendelian randomization (MR) can generate more reliable evidence with less confounding and reverse causation by using genetic variants related to the exposure or drug target for causal inference and prediction of potential adverse outcomes for a specific drug target (10, 11). In this study, we utilized a drug target MR analysis to determine the causal relationship between GLP-1RAs and ED and employed a two-step MR to explore the pathways by which exposure affects outcome.

## 2 Methods

### 2.1 Study design

**Figure 1:**
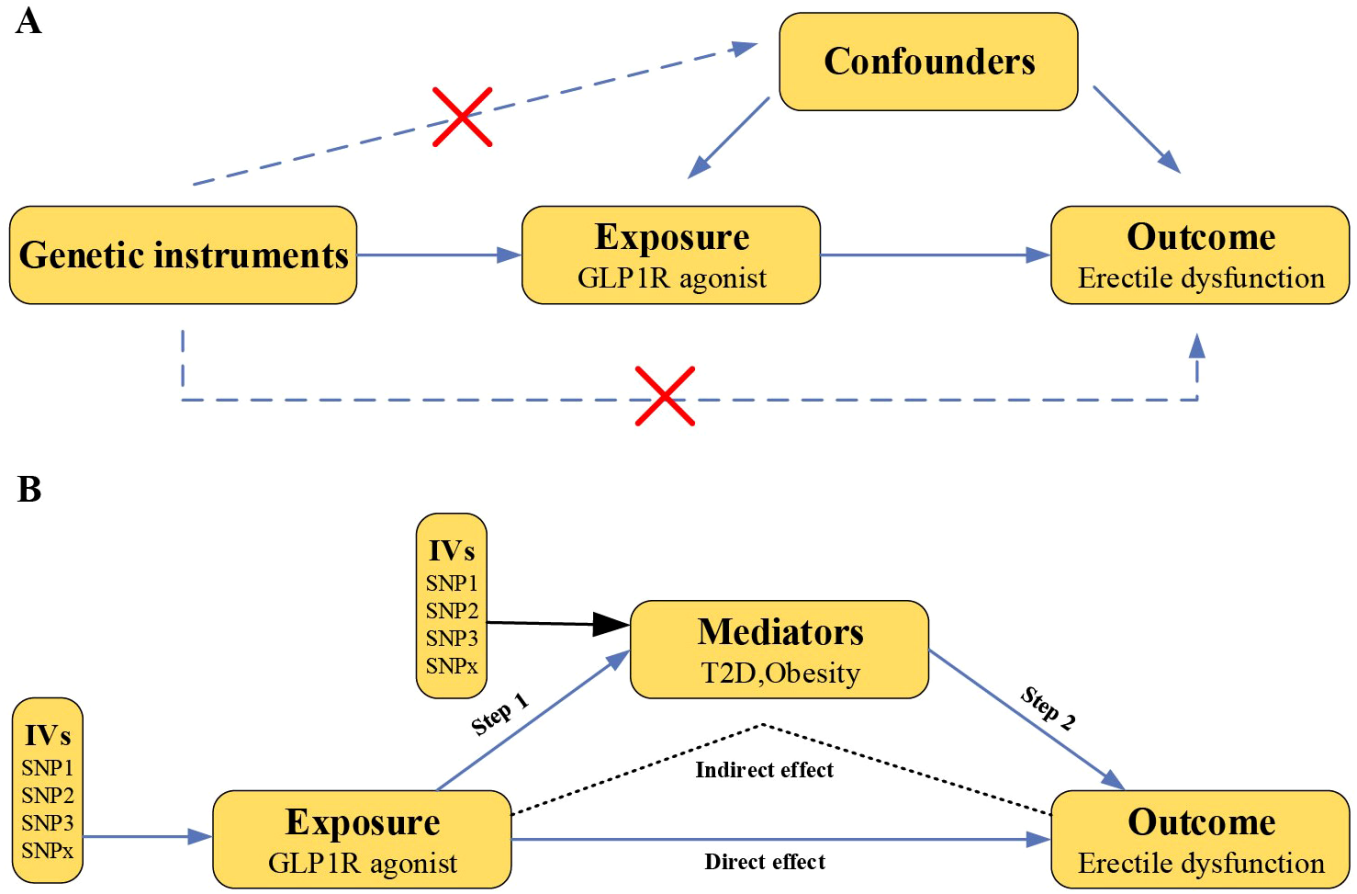
Overview of this Mendelian randomization study design. **(A)** Mendelian randomization illustration. There are three principal assumptions in Mendelian randomization design, namely the genetic instrumental variables should (1) be associated with exposure, (2) not be associated with any measured or unmeasured confounding factors and (3) be associated with outcome only via exposure. **(B)** Two-step MR analysis framework. Step 1 estimated the causal effect of the GLP1R agonist on the potential mediators, and step 2 assessed the causal effect of the mediators on erectile dysfunction risk. “Indirect effect” indicates the effect of GLP1R agonist on erectile dysfunction risk through the mediator. “Direct effect” indicates the remaining effect of the GLP1R agonist on erectile dysfunction that acts through pathways other than the specified mediator or set of mediators. (GLP1R, glucagon-like peptide-1 receptor; IVs, instrumental variables).

The drug target MR analysis was employed to investigate whether GLP-1RAs causally link to the risk of ED (**Figure 1**). First, we validated genetic instruments by analyzing the effect of GLP-1RAs on T2D and obesity as positive controls. Second, we utilized MR analyses with ED as an outcome. Third, the two-step MR was performed to reveal mediating effect in the causal relationship between GLP-1RAs and ED. Fourth, the robustness of our results was evaluated via comprehensive sensitivity analyses. Finally, we assessed the impact of potential confounders on the results by Two-Sep cis-MR (TSCMR) (12). The genetic variants for MR analyses adhered to three principal criteria (10): association with the exposure, no association with confounders, and influence the outcome only through the exposure. All data in this study are publicly available genome-wide association study (GWAS) statistical abstracts and therefore do not require additional ethical approval or informed consent.

### 2.2 Data sources

To avoid participants overlap, the GWAS for exposure and outcome were derived from different cohorts (**Supplementary Tables 1**, **2**). The cis-expression quantitative trait locus (cis-eQTL) for drugs target genes (GLP1R) were obtained from the eQTLGen Consortium (https://eqtlgen.org/) (13), which analyzed gene expression in 31,684 blood samples from 37 datasets (mainly European ancestry). We utilized summary statistics of T2D (cases/controls: 65,085/335,112) and obesity (cases/controls: 23,971/388,084) from the FinnGen (14). All cases were clinically diagnosed and genome-wide association analyses for each trait were adjusted for sex, age, genetic components, and genotyping batch (14). Summary statistics for ED were retrieved from the recent GWAS in the European population from three cohorts (UK Biobank, Estonian Genome Center of the University of Tartu and Partners HealthCare Biobank) which included a total of 6,175 cases and 217,630 controls (15). The cases were confirmed according to the code of ICD 10 (N48.4 and F52.2), medical history (medication and surgery for ED), or self-reporting. The METAL software was applied for meta-analysis and genome-wide association analyses were adjusted for age and principal components.

### 2.3 Instrumental variables selection

The instrumental variables (IVs) were retrieved through the following process:

(1) To ensure the strong association between single nucleotide polymorphisms (SNPs) and the expression of GLP1R gene, we only selected SNPs with *P* value<5×10^–8^ and adjusted *P* value<0.05 in the GLP1R region (2). We searched for the SNPs selected in the first step in the GWAS Catalog database (https://www.ebi.ac.uk/gwas/home) to avoid them being associated with other genes or phenotypes (**Supplementary Table 3** contains the deleted SNPs) (3). the SNPs were clumped for linkage disequilibrium (clumping R^2^ = 0.01) (4) Calculated the F-statistics (beta^2^/SE^2^) of SNP and excluded SNP with values less than 10 to avoid weak instrumental variable bias (5). Assessed the association of genetically proxied GLP-1RAs with T2D and obesity.

### 2.4 Statistical analysis

#### 2.4.1 MR analysis

Before MR analysis, we harmonized exposure and outcome data by aligning effect alleles to the forward strand, discarding palindromic variants. MR analysis primarily used the inverse variance weighted (IVW) method, the most effective method, supplemented by weighted-median, MR-Egger, and MR-PRESSO approaches. IVW combines SNP-specific estimates calculated using Wald ratios, assuming no directional pleiotropy of each SNP (16). The weighted median provides reliable estimates if over 50% of IVs are valid (17). MR-Egger yields directional horizontal pleiotropy corrected causal estimates but with a wider confidence interval (18). MR-PRESSO corrects for pleiotropy by removing outliers, ensuring robust causal inference (19).

#### 2.4.2 Mediation analysis

A two-step MR analysis (product of coefficients method) was applied to assess mediation (20). In the first step, genetically proxied GLP-1RAs were used to estimate the causal effect of the exposure on the potential mediators (Beta 1). In the second step, genetic instruments for the potential mediators were used to assess the causal effect of the potential mediators on ED risk (Beta 2). The mediation proportion of each mediator in the total effect of GLP-1RAs on ED was calculated by [(Beta 1 × Beta 2)/Beta 3], and Beta 3 is the total effect, an estimate of the GLP-1RAs on ED obtained from the previously described MR analysis. Finally, we applied the delta method to derive the 95% confidence intervals (CIs) of the mediation proportions (21). In addition, we used the “difference in coefficients method” as an additional sensitivity analysis to provide a complementary analysis of the mediation ratios (22). The methodology is further illustrated in **Supplementary Figure 1**.

#### 2.4.3 Adjustment for potential confounders

Considering that aging, BMI, smoking, drinking, cardiovascular disease (CVD), hypertension, metabolic syndrome, dyslipidemia and some medications are potential risk factors for ED (23, 24), we included these factors for further analysis. Similar to previous studies (12, 25), we used the TSCMR to adjust for these confounders to assess the effect of GLP-1RAs on ED. TSCMR differs from two-step MR in that the latter uses two MR estimates to calculate the indirect effect of the exposure on the outcome, whereas TSCMR uses one MR estimate and two variant phenotype estimates to assess the direct effect of the exposure on the outcome and attenuate confounders bias (12). **Supplementary Table 4** provides detailed information on the GWAS of potential confounders.

#### 2.4.4 Sensitivity analysis

A series of sensitivity analyses were performed to assess the robustness of the results. First, Cochran’s Q test detected heterogeneity (26). Then, the MR-Egger regression intercept assessed horizontal pleiotropy, indicating pleiotropy when deviating from zero (18). Lastly, a leave-one-out analysis sequentially excluded each SNP, using IVW on remaining SNPs to evaluate variant impacts (27).

Statistical analyses were conducted in R (version: 4.3.0) with TwoSampleMR (version: 0.5.7), MRPRESSO (version: 1.0) and TwoStepCisMR (version: 0.0.0.9) packages. Effect estimates are reported as odds ratios (OR) with 95% CI.

## 3 Results

### 3.1 Baseline characteristics

**Figure 2:**
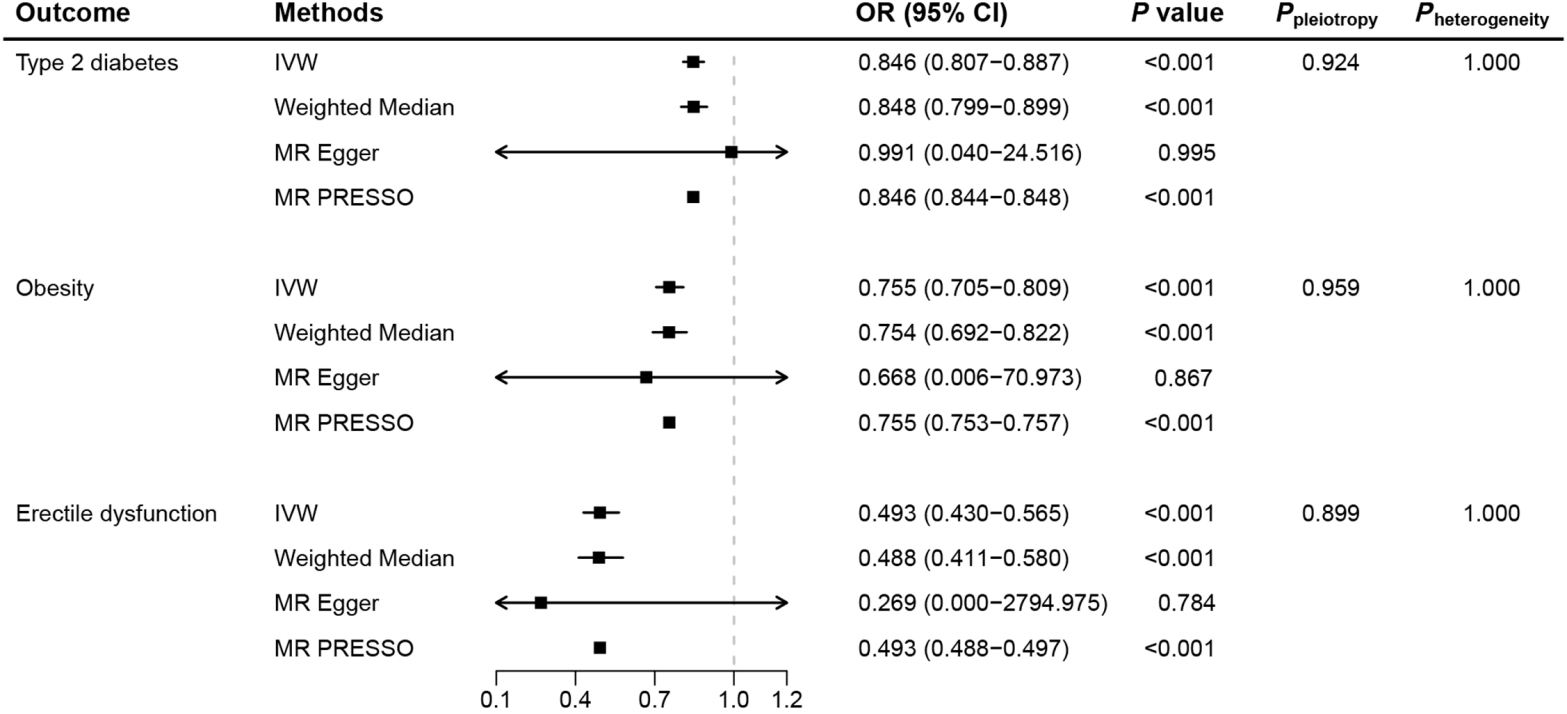
Mendelian randomization results for genetically proxied glucagon-like peptide-1 receptor agonist. (MR, Mendelian randomization; IVW, inverse-variance weighted; OR, odds ratio; CI, confidence interval).

We selected 22 significant cis-eQTL SNPs from eQTLGen as IVs for the drug target GLP1R gene, with F-statistics ranging from 47.19 to 52.93 (**Supplementary Table 5**), indicating robust instrument strength. Moreover, positive control results demonstrated that GLP-1RAs significantly reduced the risk of T2D (OR: 0.846, 95% CI: 0.807−0.887, *P*<0.001) and obesity (OR: 0.755, 95% CI: 0.705−0.809, *P*<0.001) (**Figure 2**; **Supplementary Figure 2** **–** **5**), further assuring the validity of the identified genetic instruments.

### 3.2 MR analysis of GLP-1RAs on ED

As the primary method in MR analysis, IVW estimates suggested that GLP-1RAs reduced the risk of ED (OR: 0.493, 95% CI: 0.430−0.565, *P*<0.001), corroborated by consistent results from the weighted median and MR-PRESSO methods (**Figure 2**). Individual SNP effects and combined effects from each MR method were visualized in the scatter plot (**Supplementary Figure 6**). Further sensitivity analyses showed the absence of pleiotropy (*P*_pleiotropy_=0.899) and heterogeneity (*P*_heterogeneity_=1.000), again demonstrating the robustness of the conclusions. Leave-one-out plots suggested that the associations were unlikely to be driven by certain extreme SNPs (**Supplementary Figure 7**).

### 3.3 Mediation analysis of GLP-1RAs on ED

We conducted a two-step MR analysis to investigate the mediating pathway from GLP-1RAs to ED via two GLP-1RAs related indications, including T2D and obesity. In addition, we also analyzed eleven other candidate mediators (**Supplementary Table 4**).

**Figure 3:**
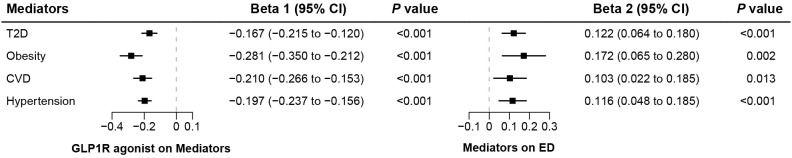
The results of two-step Mendelian randomization analysis. (Beta 1 represented the causal effect of the GLP1R agonist on the mediators, and Beta 2 represented the causal effect of the mediators on ED risk).

**Figure 4:**
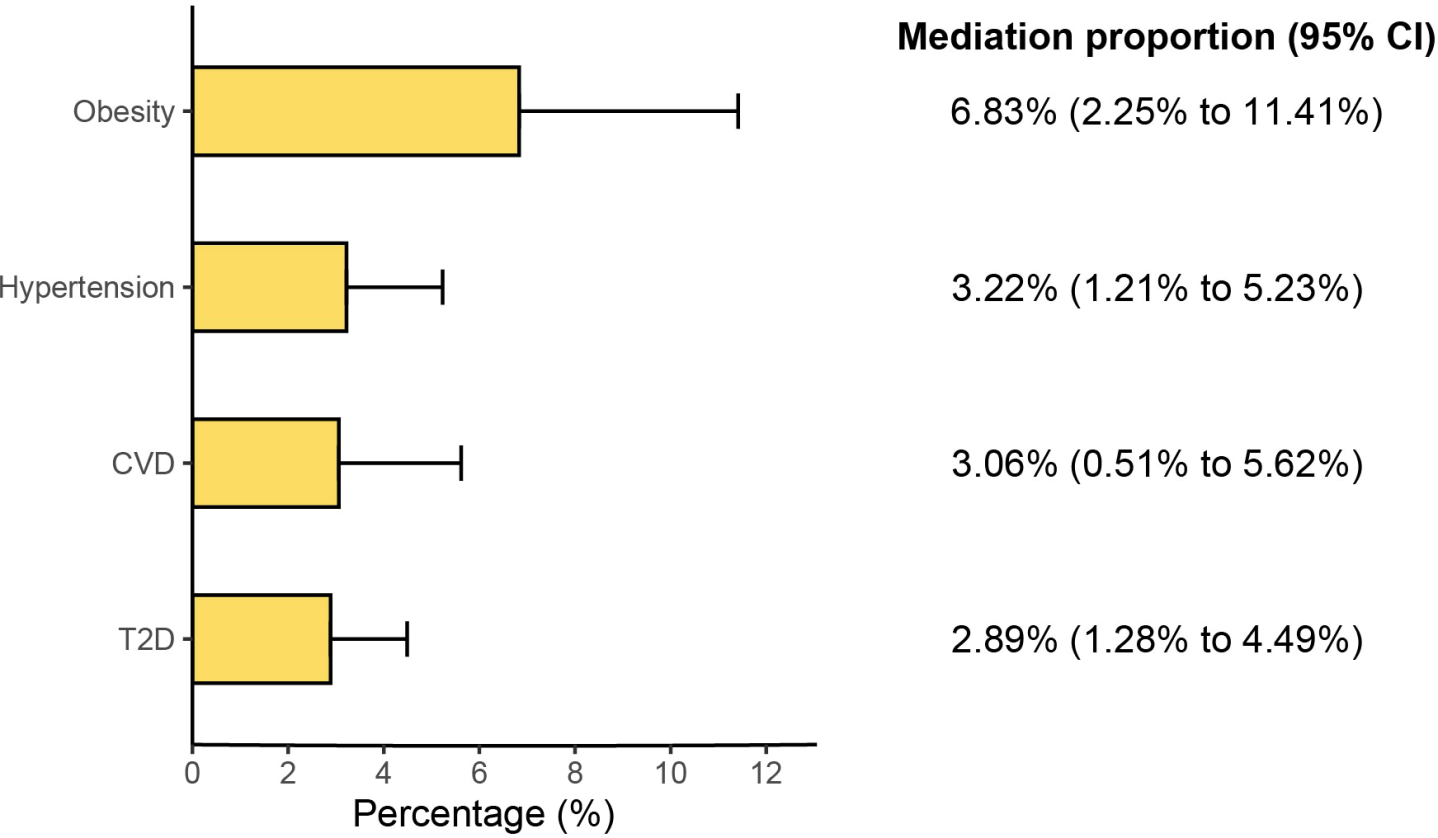
Results of the mediation proportion. (CI, confidence interval; T2D, type 2 diabetes; CVD, cardiovascular disease).

In the first step, genetic instruments for GLP-1RAs were used to estimate the causal effect of the exposure on the T2D and obesity. We identified that GLP-1RAs reduced the risk of T2D (Beta: −0.167, 95% CI: −0.215 to −0.120, *P*<0.001) and obesity (Beta: −0.281, 95% CI: −0.350 to −0.212, *P*<0.001) (**Figure 3**). In the second step, we assessed the causal effect of the mediators on ED risk using genetic instruments for the T2D and obesity (**Supplementary Tables 6**, **7**). We found causal evidence for effects of T2D (Beta: 0.122, 95% CI: 0.064 to 0.180, *P*<0.001) and obesity (Beta: 0.172, 95% CI: 0.065 to 0.280, *P*= 0.002) on ED (**Figure 3**). The weighted median and MR-PRESSO methods provided consistent findings (**Supplementary Table 8**). Finally, we estimated the indirect effect of GLP-1RAs on ED via T2D and found that the mediation effect of T2D was -0.020 (95% CI: -0.032 to -0.009; *P*<0.001) with a mediated proportion of 2.89% (95% CI: 1.28% to 4.49%) (**Figure 4**). Likewise, the mediation effect of obesity was -0.048 (95% CI: -0.081 to -0.016; *P*=0.003) with a mediated proportion of 6.83% (95% CI: 2.25% to 11.41%) (**Figure 4**). Furthermore, we also found that the protective effect of GLP-1RAs against ED is partly mediated by reducing hypertension (mediated proportion: 3.22%, 95% CI: 1.21% to 5.23%) and CVD (mediated proportion: 3.06%, 95% CI: 0.51% to 5.62%) (**Figures 3**, **4**). The results showed that the “difference in coefficients method” is very similar to the results of the “two-step MR (product of coefficients method)”, indicating that the results are robust (**Supplementary Table 9**).

### 3.4 Adjusted estimates

When adjusting for potential pleiotropic bias in the effect of GLP-1RAs on ED using TSCMR, the findings of the main analyses did not change substantially (**Supplementary Table 10**). This suggests that our results are unlikely to be affected by unreported confounders.

## 4 Discussion

In this study, by using genetic variants as unconfounded proxies for the GLP-1RAs, we attempted to disentangle relationships between GLP-1RAs and ED risk. We observed evidence indicating a protective causal effect of GLP-1RAs on ED risk. Our results were essentially robust to the different MR methods that make different assumptions about horizontal pleiotropy, suggesting that horizontal pleiotropy is unlikely to adequately explain our results. We also conducted a mediation analysis to estimate potential mediators and showed that the effect of GLP-1RAs on ED risk was partially mediated by T2D, obesity, hypertension and CVD.

Only a few studies have evaluated the association between GLP-1RAs and ED. Specifically, a recent retrospective study showed that GLP-1RAs plus metformin were superior to metformin alone in improving ED, regardless of different background characteristics of patients (6). In addition, another exploratory analysis has also suggested that long-term use of dulaglutide (belongs to GLP-1RAs) might reduce the incidence of moderate or severe ED in men with type 2 diabetes who have undergone a previous cardiovascular event or cardiovascular risk factors (7). However, up to now, the direct effect of GLP-1RAs on ED remains unknown, especially in populations without cardiovascular disease or T2D but in need of GLP-1RAs for weight management. Our MR analysis provided causal evidence that GLP-1RAs reduced the risk of ED, which offered some strategies for preventing ED in T2D and obese individuals.

**Figure 5:**
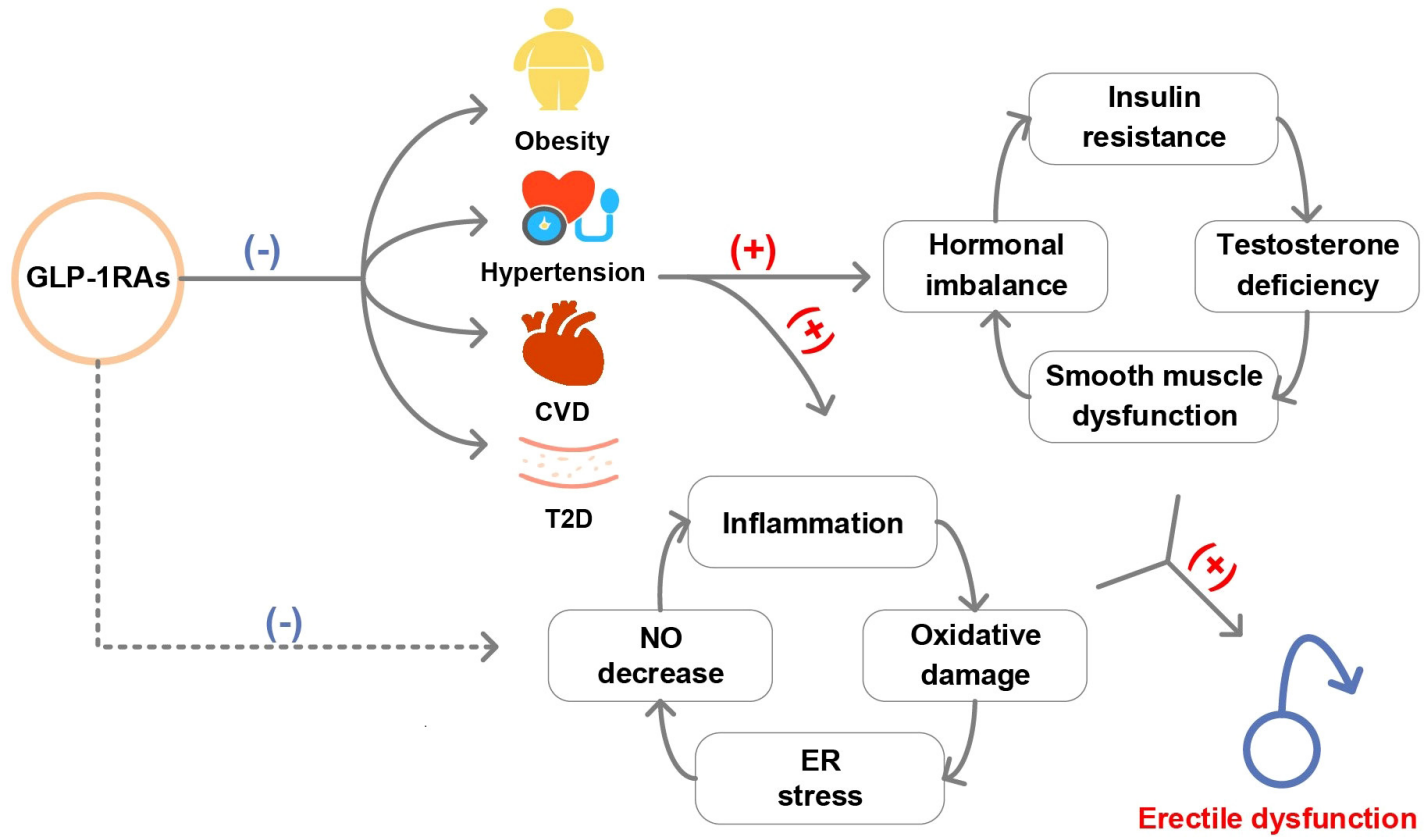
Graphical abstract of this study. (T2D, type 2 diabetes; CVD, cardiovascular disease; NO, nitric oxide; ER, endoplasmic reticulum).

Multiple biological mechanisms are hypothesized to mediate the potentially beneficial role of GLP-1RAs in ED development (**Figure 5**), such as induction of positive vascular effects and weight loss. On the one hand, GLP-1 treatment enhanced meal-induced endogenous secretion of insulin and inhibits glucagon secretion, thereby improving glucose homeostasis (28). Many clinical trials have shown that GLP-1RAs have a cardiovascular protective effect by regulating glucose (29–31), which played a preventive role for ED caused by endothelial dysfunction (32). This was confirmed by our mediation analysis that GLP-1RAs reduced the risk of ED by reducing the occurrence of T2D. On the other hand, the potent weight loss effect of GLP-1RAs contributed to the prevention of ED. Obesity can lead to ED caused by hormonal imbalance, insulin resistance, decreased testosterone circulation and worsening of testosterone deficiency (33, 34). Effective weight loss in GLP-1RAs users could alleviate this imbalance, thereby providing a protective effect for ED. Similarly, our analysis confirmed the mediation effects of obesity between GLP-1RAs and ED. In addition, consistent with previous studies, we also found that GLP-1RAs may reduce the risk of ED through positive cardiovascular effects (reduce the risk of hypertension and CVD) (35, 36).

Another mechanism theory for GLP-1RAs prevented ED might be an effect unrelated to glycemic control. In rodent studies, GLP-1RAs reduced oxidative stress and autophagy, smooth muscle dysfunction, enhanced nitric oxide-mediated diastole, protected endothelial cell function in the corpus cavernosum and reduced proinflammatory cytokine expression in the testes (37–40). However, responses in humans potentially differ from those in animals and future vigorous studies are needed to investigate the mediating role of these mechanisms between GLP-1RAs and ED.

Diabetes, obesity and ED are closely linked (41). On the one hand, the chronic inflammatory status induced by diabetes and obesity can stimulate the endothelium, leading to the uncoupling of nitric oxide synthase and endothelial dysfunction, which causes an unsuccessful erection (41–43). On the other hand, diabetes and obesity can lead to excessive production of reactive oxygen species (ROS) in mitochondria and cause oxidative stress, which not only exacerbates the damage caused by chronic inflammation, but also directly damages the endothelial cells of the penile blood vessels (41). As a drug that has emerged to treat T2D and obesity (5, 44), elucidating the effect of GLP-1RAs on the ED in the specific population may help expand the indications or contraindications of GLP-1RAs.

The potential significance of our study is that it provides some evidence of the safety of GLP-1RAs on male sexual function, and suggests that GLP-1RAs might be a potential preventive drug for ED, especially in people with diabetes or obesity. Most current treatments for ED are not cause-specific and provide only a transient effect (e.g. phosphodiesterase-5 inhibitor drugs) (45, 46). Our results support the possibility that GLP-1RAs may improve male sexual function by reducing the causes of ED such as obesity. However, further research is needed to confirm the actual effect of GLP-1RAs on improving penile endothelial dysfunction and on different degrees of ED.

The present study has the following advantages. First, we explored the causal association of GLP-1RAs and ED by using an MR design, which was less susceptible to the effects of confounders, reverse causation, and various biases (10, 47, 48). Second, complementary methods and sensitivity analyses such as weighted median, MR-Egger, MR-PRESSO, Cochran’s Q test, MR-Egger intercept and leave-one-out analysis were performed to ensure the consistency and robustness of the results.

Despite these strengths, some limitations should be acknowledged. Above all, as with all MR studies, pleiotropy effects in the MR setting are challenging (10). We have used methods such as the MR-PRESSO test and sensitivity analyses to assess pleiotropy. All of the above tests showed robust results. Then, all data involved in the analyses were obtained from individuals of European ancestry, restricting the generalizability of our findings to other ethnic groups. Next, due to the limited sample size of the data, further research in a larger sample population is necessary. Furthermore, although the GWAS data used adopted the same criteria to measure phenotypes, our conclusions still should be generalized with caution to the general population until there is sufficient evidence, considering the effects of selection bias and remaining possible measurement bias (49). Fortunately, due to the large size and diversity of biobanks, these scientific inferences still provide some insight when generalized to the general population (49). Finally, the dose-response of the genetically proxied GLP-1RAs may differ from that of the patient, so further studies are required to confirm this, and these findings should not promote the indiscriminate use of GLP-1RAs.

## 5 Conclusion

In conclusion, we provided evidence that genetically proxied GLP-1RAs were associated with a reduced risk of ED, and to a lesser extent, T2D, obesity, hypertension and CVD mediated this effect. Nevertheless, the potential implications of our results for ED prevention policies required validation in further clinical randomized controlled trials.

## Data Availability

Publicly available datasets were analyzed in this study. This data can be found here: https://eqtlgen.org/cis-eqtls.html https://molgenis26.gcc.rug.nl/downloads/eqtlgen/cis-eqtl/2019-12-11-cis-eQTLsFDR-ProbeLevel-CohortInfoRemoved-BonferroniAdded.txt.gz; https://gwas.mrcieu.ac.uk/datasets/ebi-a-GCST006956/, https://www.finngen.fi/en, https://storage.googleapis.com/finngen-public-data-r10/summary_stats/finngen_R10_T2D.gz; https://storage.googleapis.com/finngen-public-data-r10/summary_stats/finngen_R10_E4_OBESITY.gz.
