# Supplementary material for: Glucagon-like peptide-1 receptor agonists and the risk of erectile dysfunction: a drug target Mendelian randomization study
Source: Front Endocrinol (Lausanne). 2024 Nov 13;15:1448394. doi: 10.3389/fendo.2024.1448394 (PMC11600104; doi:10.3389/fendo.2024.1448394)
Supplement: Supplementary file 1 [file DataSheet1.docx]

**Supplementary Tables and Figures**

Supplementary Table 1 p2

Supplementary Table 2 p3

Supplementary Table 3 p4

Supplementary Table 4 p5

Supplementary Table 5 p6

Supplementary Table 6 p7

Supplementary Table 7 p11

Supplementary Table 8 p12

Supplementary Table 9 p13

Supplementary Table 10 p14

Supplementary Figure 1 p15

Supplementary Figure 2 p16

Supplementary Figure 3 p17

Supplementary Figure 4 p18

Supplementary Figure 5 p19

Supplementary Figure 6 p20

Supplementary Figure 7 p21

**Supplementary Table 1. Information about each participating cohort in eQTLGen Consortium.**

| **Cohort** | **Population** | **Samples** |
| --- | --- | --- |
| CODAM | European (Netherlands) | 186 |
| GoNL | European (Netherlands) | 85 |
| Lifelines Deep | European (Netherlands) | 1114 |
| Leiden Longevity Study | European (Netherlands) | 432 |
| Leiden Longevity Study | European (Netherlands) | 265 |
| NTR | European (Netherlands) | 651 |
| NTR | European (Netherlands) | 147 |
| PAN | European (Netherlands) | 175 |
| Rotterdam Study | European (Netherlands) | 776 |
| EGCUT 2 | European (Estonia) | 508 |
| CARTaGENE | Predominantly European | 634 |
| CARTaGENE | Predominantly European | 191 |
| DGN | European | 922 |
| GTEx | Predominantly European | 336 |
| BEST | Bangladeshi | 1404 |
| BSGS | Northern European | 329 |
| Cardiology | Predominantly European | 134 |
| CHDWB | Predominantly European | 384 |
| DILGOM | European (Finland) | 498 |
| EGCUT 1 | European (Estonia) | 818 |
| EGCUT 2 | European (Estonia) | 77 |
| Fehrmann | European (United Kingdom and Netherlands) | 214 |
| Fehrmann | European (United Kingdom and Netherlands) | 1227 |
| HVH | American | 40 |
| HVH | American | 59 |
| InCHIANTI | European (Italy) | 609 |
| KORA F4 | European (Germany) | 952 |
| LIFE Adult | European (Germany) | 1978/2456 |
| LIFE Heart | European (Germany) | 2106/4285 |
| Morocco | Arabs and Amazighs | 175 |
| Rotterdam Study | European (Netherlands) | 749 |
| SHIP-TREND | European (North-east of Germany) | 955 |
| SSIC | Chinese | 115 |
| Sorbs | European (Sorbs slavonic minority from Germany) | 513 |
| Young Finns Study | European (Finland) | 1428 |
| FHS | American | 5075/1549 |
| NTR-NESDA | European (Netherlands) | 2767 |

**Supplementary Table 2. Information about each participating cohort of erectile dysfunction.**

| **Cohort** | **Population** | **Samples** |
| --- | --- | --- |
| UK Biobank | European (UK) | 199,352 |
| PHB | European (USA) | 7,666 |
| EGCUT | European (Republic of Estonia) | 16,787 |

UKBB: UK Biobank;

PHB: Partners HealthCare Biobank;

EGCUT: Estonian Genome Center of the University of Tartu.

**Supplementary Table 3. Single nucleotide polymorphisms (SNPs) deleted due to association with other genes or phenotypes.**

| **SNP** | **Trait** |
| --- | --- |
| rs10305420 | smoking initiation |
| rs1412265 | smoking initiation |
| rs10305420 | type 2 diabetes mellitus |
| rs2281342 | type 2 diabetes mellitus |
| rs1738249 | vascular dementia |
| rs2268657 | body mass index |

**Supplementary Table 4. Information on the GWAS of potential confounders.**

| **Confounders** | **sample size** | **Ancestry** | **Consortium** | **PMID** |
| --- | --- | --- | --- | --- |
| Human aging | 154,478 | European | Meta | 37118362 |
| BMI | 290,820 | European | FinnGen | 36653562 |
| Smoking | 2,669,029 | European | Meta | 36477530 |
| Drinking | 2,669,029 | European | Meta | 36477530 |
| CVD | 412,181 | European | FinnGen | 36653562 |
| Hypertension | 412,113 | European | FinnGen | 36653562 |
| Metabolic syndrome | 412,181 | European | FinnGen | 36653562 |
| Hyperlipidemia | 365,889 | European | FinnGen | 36653562 |
| Antihypertensives | 152,380 | European | UK Biobank | 31015401 |
| Immunosuppressants | 272,602 | European | UK Biobank | 31015401 |
| NSAIDs | 164,520 | European | UK Biobank | 31015401 |

BMI: body mass index, CVD: cardiovascular disease;

NSAIDs: nonsteroidal anti-inflammatory Drugs.

**Supplementary Table 5. Genetic instrumental variables of Glucagon-like peptide-1 receptor agonists (GLP-1RAs) for the drug target Mendelian randomization.**

| **SNP** | **Effect allele** | **Other allele** | **Beta** | **SE** | **F-statistics** | ***P* value** |
| --- | --- | --- | --- | --- | --- | --- |
| rs1678682 | G | A | -0.05700 | 0.00830 | 47.19 | 6.45E-12 |
| rs9394544 | A | G | -0.05794 | 0.00831 | 48.66 | 3.05E-12 |
| rs1678692 | C | T | -0.05825 | 0.00832 | 49.05 | 2.50E-12 |
| rs9349100 | T | C | -0.05815 | 0.00829 | 49.15 | 2.36E-12 |
| rs1678691 | C | T | -0.05847 | 0.00832 | 49.43 | 2.06E-12 |
| rs1678693 | C | T | -0.05928 | 0.00832 | 50.77 | 1.04E-12 |
| rs1738212 | C | A | -0.05961 | 0.00832 | 51.33 | 7.80E-13 |
| rs1738199 | A | G | -0.05947 | 0.00830 | 51.38 | 7.61E-13 |
| rs984524 | G | A | -0.05967 | 0.00832 | 51.42 | 7.43E-13 |
| rs1678712 | G | A | -0.05970 | 0.00832 | 51.48 | 7.24E-13 |
| rs6919465 | G | A | -0.05972 | 0.00832 | 51.51 | 7.11E-13 |
| rs9380787 | G | T | -0.05932 | 0.00826 | 51.52 | 7.07E-13 |
| rs1678706 | G | A | -0.05977 | 0.00832 | 51.59 | 6.83E-13 |
| rs1678707 | G | A | -0.05982 | 0.00832 | 51.69 | 6.49E-13 |
| rs1738203 | A | G | -0.05972 | 0.00830 | 51.73 | 6.38E-13 |
| rs1678695 | A | G | -0.06000 | 0.00832 | 51.99 | 5.58E-13 |
| rs1678702 | C | A | -0.06003 | 0.00832 | 52.04 | 5.44E-13 |
| rs1678700 | G | A | -0.06004 | 0.00832 | 52.05 | 5.39E-13 |
| rs1629877 | C | A | -0.06016 | 0.00832 | 52.28 | 4.82E-13 |
| rs1678696 | G | A | -0.06028 | 0.00832 | 52.47 | 4.37E-13 |
| rs1678697 | G | A | -0.06028 | 0.00832 | 52.48 | 4.34E-13 |
| rs1678701 | A | G | -0.06056 | 0.00832 | 52.93 | 3.45E-13 |

SNP: single nucleotide polymorphism, SE: standard error.

**Supplementary Table 6. Genetic instrumental variables of type 2 diabetes.**

| **SNP** | **Effect allele** | **Other allele** | **Beta** | **SE** | **F-statistics** | ***P* value** |
| --- | --- | --- | --- | --- | --- | --- |
| rs81204 | C | T | -0.04125 | 0.00756 | 29.76 | 4.89E-08 |
| rs6944766 | A | G | -0.04557 | 0.00835 | 29.82 | 4.75E-08 |
| rs118158020 | A | G | -0.09422 | 0.01724 | 29.86 | 4.64E-08 |
| rs34029654 | A | G | -0.05467 | 0.01000 | 29.87 | 4.62E-08 |
| rs62484946 | C | T | 0.08348 | 0.01526 | 29.92 | 4.50E-08 |
| rs55877252 | G | T | -0.07272 | 0.01327 | 30.04 | 4.23E-08 |
| rs77789961 | T | C | 0.08536 | 0.01553 | 30.22 | 3.86E-08 |
| rs554186429 | C | G | -0.07985 | 0.01450 | 30.31 | 3.67E-08 |
| rs35005436 | T | C | -0.05641 | 0.01024 | 30.35 | 3.60E-08 |
| rs113933090 | A | G | 0.11444 | 0.02075 | 30.43 | 3.47E-08 |
| rs150654093 | A | G | 0.12794 | 0.02317 | 30.49 | 3.36E-08 |
| rs749773 | C | T | 0.04041 | 0.00731 | 30.53 | 3.30E-08 |
| rs13067541 | T | C | -0.06931 | 0.01251 | 30.70 | 3.02E-08 |
| rs62442903 | A | G | -0.04128 | 0.00743 | 30.83 | 2.81E-08 |
| rs111716216 | T | C | -0.04601 | 0.00825 | 31.12 | 2.42E-08 |
| rs7631359 | G | T | -0.05881 | 0.01052 | 31.23 | 2.29E-08 |
| rs10487174 | T | C | -0.03717 | 0.00664 | 31.30 | 2.21E-08 |
| rs11074585 | G | A | -0.05567 | 0.00994 | 31.40 | 2.10E-08 |
| rs6850761 | C | A | -0.03829 | 0.00682 | 31.55 | 1.95E-08 |
| rs2733269 | G | A | 0.03711 | 0.00660 | 31.64 | 1.86E-08 |
| rs1265089 | A | G | 0.04417 | 0.00782 | 31.90 | 1.63E-08 |
| rs12133396 | A | G | -0.04297 | 0.00759 | 32.04 | 1.51E-08 |
| rs10983127 | C | T | -0.04805 | 0.00848 | 32.13 | 1.44E-08 |
| rs34312169 | A | C | 0.04978 | 0.00870 | 32.72 | 1.06E-08 |
| rs11774221 | G | A | -0.04603 | 0.00804 | 32.74 | 1.06E-08 |
| rs72683004 | A | C | -0.04211 | 0.00732 | 33.08 | 8.83E-09 |
| rs61775082 | T | C | -0.05616 | 0.00972 | 33.42 | 7.43E-09 |
| rs187642402 | C | T | 0.08572 | 0.01481 | 33.51 | 7.10E-09 |
| rs12001437 | C | T | 0.03974 | 0.00685 | 33.61 | 6.72E-09 |
| rs35446205 | G | C | -0.06774 | 0.01168 | 33.64 | 6.63E-09 |
| rs9913260 | A | G | 0.04920 | 0.00847 | 33.75 | 6.27E-09 |
| rs12440774 | C | G | 0.03854 | 0.00663 | 33.76 | 6.24E-09 |
| rs73610830 | G | C | -0.04176 | 0.00718 | 33.79 | 6.14E-09 |
| rs2521248 | T | C | 0.03933 | 0.00676 | 33.82 | 6.05E-09 |
| rs7306319 | G | A | -0.04502 | 0.00769 | 34.30 | 4.74E-09 |
| rs11642255 | T | C | -0.09046 | 0.01544 | 34.32 | 4.68E-09 |
| rs9855524 | T | A | 0.04341 | 0.00737 | 34.64 | 3.96E-09 |
| rs4374177 | G | A | -0.06098 | 0.01034 | 34.76 | 3.72E-09 |
| rs7226371 | G | A | 0.05089 | 0.00862 | 34.86 | 3.54E-09 |
| rs112016043 | G | T | 0.12002 | 0.02024 | 35.16 | 3.04E-09 |
| rs2306141 | G | C | 0.06579 | 0.01108 | 35.23 | 2.93E-09 |
| rs1665908 | T | A | -0.03982 | 0.00671 | 35.24 | 2.91E-09 |
| rs17039732 | A | T | 0.08958 | 0.01508 | 35.31 | 2.82E-09 |
| rs1798090 | C | T | -0.03949 | 0.00662 | 35.55 | 2.49E-09 |
| rs61887147 | A | C | 0.04655 | 0.00780 | 35.59 | 2.44E-09 |
| rs11576360 | G | A | 0.05060 | 0.00846 | 35.74 | 2.26E-09 |
| rs13288841 | A | G | 0.04040 | 0.00676 | 35.75 | 2.24E-09 |
| rs36106955 | T | C | 0.04802 | 0.00803 | 35.75 | 2.24E-09 |
| rs10404726 | T | C | -0.03925 | 0.00656 | 35.79 | 2.20E-09 |
| rs9818870 | T | C | 0.06318 | 0.01055 | 35.87 | 2.11E-09 |
| rs7146599 | A | G | 0.03939 | 0.00657 | 35.91 | 2.07E-09 |
| rs2589408 | G | A | -0.03980 | 0.00662 | 36.11 | 1.86E-09 |
| rs607409 | T | A | 0.04057 | 0.00674 | 36.20 | 1.78E-09 |
| rs2812535 | A | G | 0.04291 | 0.00713 | 36.25 | 1.74E-09 |
| rs17383290 | G | A | -0.04489 | 0.00746 | 36.26 | 1.73E-09 |
| rs12420590 | G | C | 0.04583 | 0.00760 | 36.33 | 1.66E-09 |
| rs56901542 | G | A | -0.04392 | 0.00728 | 36.36 | 1.64E-09 |
| rs7948345 | G | C | -0.05602 | 0.00924 | 36.74 | 1.35E-09 |
| rs3096301 | T | C | 0.04153 | 0.00684 | 36.91 | 1.24E-09 |
| rs7140574 | A | G | 0.04030 | 0.00661 | 37.14 | 1.10E-09 |
| rs78486128 | A | G | 0.06535 | 0.01071 | 37.21 | 1.06E-09 |
| rs73224247 | C | T | -0.06775 | 0.01110 | 37.26 | 1.03E-09 |
| rs9350408 | T | C | -0.04010 | 0.00657 | 37.27 | 1.03E-09 |
| rs618652 | G | T | -0.04012 | 0.00657 | 37.30 | 1.01E-09 |
| rs4289073 | T | G | -0.04090 | 0.00662 | 38.21 | 6.34E-10 |
| rs62092443 | T | C | 0.07006 | 0.01131 | 38.37 | 5.84E-10 |
| rs7661876 | G | T | 0.04090 | 0.00656 | 38.83 | 4.62E-10 |
| rs11169182 | T | C | 0.04104 | 0.00656 | 39.10 | 4.02E-10 |
| rs897558 | G | A | 0.04106 | 0.00656 | 39.17 | 3.89E-10 |
| rs74628648 | T | C | -0.07726 | 0.01234 | 39.20 | 3.83E-10 |
| rs79400013 | A | G | -0.15589 | 0.02489 | 39.24 | 3.74E-10 |
| rs4273712 | G | A | 0.04602 | 0.00734 | 39.29 | 3.65E-10 |
| rs10775406 | G | A | 0.04681 | 0.00746 | 39.33 | 3.59E-10 |
| rs4681047 | G | A | -0.04143 | 0.00657 | 39.77 | 2.86E-10 |
| rs72813935 | C | T | -0.08387 | 0.01326 | 39.98 | 2.57E-10 |
| rs6918311 | G | A | -0.04196 | 0.00656 | 40.87 | 1.63E-10 |
| rs9909861 | A | C | -0.04499 | 0.00703 | 40.93 | 1.58E-10 |
| rs9371672 | G | C | 0.04443 | 0.00693 | 41.10 | 1.45E-10 |
| rs35196956 | C | T | -0.11449 | 0.01781 | 41.30 | 1.30E-10 |
| rs429150 | C | T | 0.04281 | 0.00657 | 42.49 | 7.09E-11 |
| rs35229997 | G | T | 0.04992 | 0.00765 | 42.57 | 6.81E-11 |
| rs10974438 | C | A | 0.04413 | 0.00676 | 42.57 | 6.80E-11 |
| rs17168486 | T | C | 0.05340 | 0.00818 | 42.64 | 6.59E-11 |
| rs6839635 | A | C | -0.04313 | 0.00659 | 42.87 | 5.87E-11 |
| rs7845219 | C | T | -0.04286 | 0.00654 | 42.89 | 5.78E-11 |
| rs28798715 | A | C | -0.06779 | 0.01029 | 43.36 | 4.54E-11 |
| rs12531884 | C | A | -0.04356 | 0.00658 | 43.78 | 3.68E-11 |
| rs4804833 | G | A | -0.04466 | 0.00675 | 43.81 | 3.62E-11 |
| rs141433349 | A | G | 0.12519 | 0.01886 | 44.07 | 3.17E-11 |
| rs7788808 | C | T | 0.05103 | 0.00764 | 44.59 | 2.43E-11 |
| rs4937325 | C | T | -0.04642 | 0.00694 | 44.69 | 2.31E-11 |
| rs78721871 | A | G | 0.11630 | 0.01736 | 44.90 | 2.07E-11 |
| rs66922415 | G | A | 0.05632 | 0.00838 | 45.15 | 1.82E-11 |
| rs11631200 | A | G | 0.04435 | 0.00658 | 45.38 | 1.62E-11 |
| rs61909599 | C | G | 0.07350 | 0.01091 | 45.38 | 1.62E-11 |
| rs1788817 | G | A | -0.04439 | 0.00656 | 45.74 | 1.35E-11 |
| rs4862423 | T | C | 0.04596 | 0.00676 | 46.25 | 1.04E-11 |
| rs28479795 | T | C | 0.05201 | 0.00761 | 46.67 | 8.39E-12 |
| rs6905288 | A | G | 0.04549 | 0.00665 | 46.85 | 7.66E-12 |
| rs73347525 | G | A | -0.05516 | 0.00802 | 47.26 | 6.23E-12 |
| rs387955 | T | C | 0.09990 | 0.01449 | 47.53 | 5.43E-12 |
| rs8073177 | C | T | -0.05200 | 0.00751 | 47.96 | 4.34E-12 |
| rs348330 | A | G | -0.04751 | 0.00686 | 48.00 | 4.26E-12 |
| rs17772814 | A | G | -0.07797 | 0.01119 | 48.56 | 3.20E-12 |
| rs79356898 | A | G | 0.09827 | 0.01409 | 48.65 | 3.05E-12 |
| rs1905505 | A | G | -0.05246 | 0.00746 | 49.45 | 2.04E-12 |
| rs142682088 | A | G | -0.11882 | 0.01683 | 49.85 | 1.66E-12 |
| rs3759831 | G | C | -0.05334 | 0.00754 | 50.02 | 1.52E-12 |
| rs10084393 | A | C | -0.05600 | 0.00788 | 50.48 | 1.20E-12 |
| rs488321 | C | T | -0.07964 | 0.01120 | 50.54 | 1.17E-12 |
| rs10466811 | A | G | -0.05580 | 0.00780 | 51.18 | 8.42E-13 |
| rs35658696 | G | A | 0.09928 | 0.01384 | 51.46 | 7.29E-13 |
| rs7021934 | T | A | 0.04898 | 0.00676 | 52.51 | 4.28E-13 |
| rs56384412 | G | C | 0.08179 | 0.01103 | 55.03 | 1.19E-13 |
| rs963740 | T | A | -0.05992 | 0.00807 | 55.06 | 1.17E-13 |
| rs3887925 | T | C | 0.05006 | 0.00659 | 57.75 | 2.98E-14 |
| rs144168035 | C | G | 0.13521 | 0.01744 | 60.08 | 9.13E-15 |
| rs6878122 | A | G | -0.06029 | 0.00776 | 60.34 | 7.98E-15 |
| rs1596972 | G | A | 0.05146 | 0.00660 | 60.87 | 6.11E-15 |
| rs61779309 | C | T | 0.06466 | 0.00829 | 60.88 | 6.08E-15 |
| rs8100204 | A | G | 0.07188 | 0.00899 | 63.97 | 1.26E-15 |
| rs6459735 | G | C | -0.07644 | 0.00934 | 66.93 | 2.81E-16 |
| rs72812178 | T | G | 0.07672 | 0.00937 | 67.04 | 2.66E-16 |
| rs3757840 | G | T | -0.05639 | 0.00659 | 73.22 | 1.16E-17 |
| rs2682907 | A | G | -0.05735 | 0.00668 | 73.61 | 9.53E-18 |
| rs9505086 | C | T | 0.05694 | 0.00661 | 74.17 | 7.15E-18 |
| rs739753 | T | A | -0.06693 | 0.00776 | 74.35 | 6.54E-18 |
| rs8071043 | C | T | 0.06162 | 0.00708 | 75.72 | 3.28E-18 |
| rs57898296 | G | C | 0.05983 | 0.00681 | 77.22 | 1.53E-18 |
| rs11717195 | C | T | -0.07736 | 0.00868 | 79.34 | 5.23E-19 |
| rs149991550 | T | C | 0.19255 | 0.02154 | 79.92 | 3.89E-19 |
| rs10504042 | A | G | 0.05905 | 0.00658 | 80.47 | 2.95E-19 |
| rs139722172 | G | C | -0.07654 | 0.00852 | 80.68 | 2.65E-19 |
| rs73040004 | C | T | 0.07053 | 0.00782 | 81.26 | 1.98E-19 |
| rs10408179 | C | T | -0.05995 | 0.00660 | 82.53 | 1.04E-19 |
| rs80323638 | A | G | -0.14437 | 0.01578 | 83.73 | 5.67E-20 |
| rs112650492 | G | C | 0.16025 | 0.01744 | 84.43 | 3.99E-20 |
| rs1046316 | G | A | 0.06366 | 0.00677 | 88.51 | 5.05E-21 |
| rs5215 | T | C | -0.06254 | 0.00655 | 91.05 | 1.40E-21 |
| rs77735929 | A | T | 0.15175 | 0.01582 | 92.02 | 8.57E-22 |
| rs35352848 | C | T | -0.06874 | 0.00713 | 92.97 | 5.32E-22 |
| rs849327 | G | A | -0.06674 | 0.00689 | 93.79 | 3.52E-22 |
| rs483082 | T | G | -0.07614 | 0.00783 | 94.49 | 2.46E-22 |
| rs10770143 | T | C | -0.06813 | 0.00690 | 97.58 | 5.18E-23 |
| rs66502159 | T | C | -0.11395 | 0.01130 | 101.68 | 6.54E-24 |
| rs10882099 | C | T | -0.06721 | 0.00656 | 105.00 | 1.22E-24 |
| rs114322470 | G | T | -0.23343 | 0.02251 | 107.57 | 3.35E-25 |
| rs28624681 | T | C | -0.07732 | 0.00726 | 113.34 | 1.82E-26 |
| rs56348580 | C | G | -0.07857 | 0.00734 | 114.55 | 9.86E-27 |
| rs45551238 | T | C | -0.16879 | 0.01565 | 116.36 | 3.97E-27 |
| rs11257655 | T | C | 0.08007 | 0.00741 | 116.81 | 3.15E-27 |
| rs17036160 | T | C | -0.09693 | 0.00880 | 121.41 | 3.11E-28 |
| rs2138157 | C | A | 0.07721 | 0.00684 | 127.26 | 1.63E-29 |
| rs78470967 | A | T | -0.20058 | 0.01744 | 132.27 | 1.31E-30 |
| rs7109575 | A | G | -0.09074 | 0.00778 | 135.88 | 2.12E-31 |
| rs11558471 | G | A | -0.08139 | 0.00678 | 143.95 | 3.65E-33 |
| rs10965247 | G | A | -0.11292 | 0.00930 | 147.54 | 5.98E-34 |
| rs7018475 | G | T | 0.09253 | 0.00726 | 162.50 | 3.22E-37 |
| rs11716713 | C | G | 0.09541 | 0.00706 | 182.89 | 1.13E-41 |
| rs112108223 | A | G | -0.33837 | 0.02362 | 205.28 | 1.47E-46 |
| rs10830963 | G | C | 0.10566 | 0.00680 | 241.22 | 2.13E-54 |
| rs2237897 | T | C | -0.19461 | 0.01245 | 244.27 | 4.61E-55 |
| rs6931514 | G | A | 0.11757 | 0.00692 | 288.80 | 9.09E-65 |
| rs7206629 | C | T | 0.11719 | 0.00663 | 312.29 | 6.92E-70 |
| rs35198068 | C | T | 0.25042 | 0.00797 | 986.07 | 1.00E-200 |

SNP: single nucleotide polymorphism, SE: standard error.

**Supplementary Table 7. Genetic instrumental variables of obesity.**

| **SNP** | **Effect allele** | **Other allele** | **Beta** | **SE** | **F-statistics** | ***P* value** |
| --- | --- | --- | --- | --- | --- | --- |
| rs10860990 | C | T | -0.05712 | 0.00983 | 33.74 | 6.31E-09 |
| rs10887571 | T | C | 0.05383 | 0.00957 | 31.61 | 1.88E-08 |
| rs10929985 | T | C | 0.05303 | 0.00968 | 30.00 | 4.31E-08 |
| rs10938398 | A | G | 0.08656 | 0.00953 | 82.54 | 1.04E-19 |
| rs11030104 | G | A | -0.10363 | 0.01308 | 62.75 | 2.35E-15 |
| rs11082279 | A | G | -0.05748 | 0.00985 | 34.03 | 5.42E-09 |
| rs11179203 | A | G | 0.06983 | 0.01277 | 29.91 | 4.52E-08 |
| rs12050481 | C | T | 0.05808 | 0.01001 | 33.64 | 6.64E-09 |
| rs12511535 | T | C | -0.05467 | 0.00983 | 30.90 | 2.72E-08 |
| rs12639495 | C | T | 0.08489 | 0.01249 | 46.21 | 1.06E-11 |
| rs12831874 | T | G | -0.07674 | 0.01363 | 31.70 | 1.80E-08 |
| rs13394970 | G | T | -0.05297 | 0.00958 | 30.55 | 3.26E-08 |
| rs1445594 | G | A | -0.06973 | 0.01021 | 46.64 | 8.55E-12 |
| rs147001051 | G | C | 0.12234 | 0.02068 | 35.00 | 3.30E-09 |
| rs1594904 | G | A | 0.05717 | 0.01014 | 31.77 | 1.73E-08 |
| rs17024258 | T | C | 0.11825 | 0.01964 | 36.24 | 1.74E-09 |
| rs1861410 | T | C | -0.06068 | 0.00967 | 39.40 | 3.46E-10 |
| rs2168711 | C | T | 0.10284 | 0.01202 | 73.16 | 1.20E-17 |
| rs34298980 | C | T | -0.06605 | 0.01033 | 40.87 | 1.62E-10 |
| rs34783010 | T | G | -0.07807 | 0.01104 | 50.04 | 1.51E-12 |
| rs35296418 | A | G | -0.07165 | 0.01190 | 36.26 | 1.73E-09 |
| rs3798519 | C | A | 0.09624 | 0.01144 | 70.77 | 4.01E-17 |
| rs3811405 | G | A | 0.05871 | 0.01063 | 30.48 | 3.37E-08 |
| rs4072287 | A | C | 0.06159 | 0.00977 | 39.75 | 2.88E-10 |
| rs45551238 | T | C | -0.14255 | 0.02300 | 38.42 | 5.70E-10 |
| rs5758187 | C | G | 0.06242 | 0.01095 | 32.47 | 1.21E-08 |
| rs59563471 | G | A | -0.06513 | 0.01117 | 34.00 | 5.52E-09 |
| rs62473704 | A | C | -0.10563 | 0.01179 | 80.26 | 3.28E-19 |
| rs66466969 | A | C | 0.05549 | 0.01018 | 29.74 | 4.94E-08 |
| rs6726297 | G | A | 0.06585 | 0.01104 | 35.56 | 2.47E-09 |
| rs6739303 | T | C | 0.12913 | 0.01320 | 95.74 | 1.31E-22 |
| rs6749170 | G | A | 0.05305 | 0.00964 | 30.26 | 3.78E-08 |
| rs7140259 | G | T | 0.05538 | 0.00962 | 33.14 | 8.58E-09 |
| rs7226371 | G | A | 0.07790 | 0.01242 | 39.33 | 3.57E-10 |
| rs76503812 | A | G | 0.10413 | 0.01898 | 30.09 | 4.12E-08 |
| rs8056890 | A | G | 0.06170 | 0.00976 | 39.94 | 2.61E-10 |

SNP: single nucleotide polymorphism, SE: standard error.

**Supplementary Table 8. Effect estimates of type 2 diabetes and obesity on erectile dysfunction.**

| **Exposure** | **methods** | **Beta (95% CI)** | ***P* value** | ***P*_pleiotropy_** | ***P*_heterogeneity_** |
| --- | --- | --- | --- | --- | --- |
| T2D |  |  |  |  |  |
|  | IVW | 0.122 (0.064 to 0.180) | <0.001 | 0.499 | 0.149 |
|  | Weighted Median | 0.158 (0.054 to 0.263) | 0.003 |  |  |
|  | MR Egger | 0.165 (0.025 to 0.305) | 0.022 |  |  |
|  | MR PRESSO | 0.122 (0.060 to 0.183) | <0.001 |  |  |
| Obesity |  |  |  |  |  |
|  | IVW | 0.172 (0.065 to 0.280) | 0.002 | 0.330 | 0.249 |
|  | Weighted Median | 0.163 (0.002 to 0.325) | 0.047 |  |  |
|  | MR Egger | -0.049  (-0.503 to 0.405) | 0.833 |  |  |
|  | MR PRESSO | 0.172 (0.057 to 0.288) | 0.006 |  |  |

T2D: type 2 diabetes; IVW: inverse variance weighted.

**Supplementary Table 9. Results of mediation proportions in different methodologies.**

| **Mediators** | **Product of coefficients method (two-step MR)** | **Difference in coefficients method** |
| --- | --- | --- |
| Type 2 diabetes | 2.89% | 2.94% |
| Obesity | 6.83% | 6.82% |
| CVD | 3.06% | 3.22% |
| Hypertension | 3.22% | 3.22% |

T2D: type 2 diabetes, BMI: body mass index, CVD: cardiovascular disease.

**Supplementary Table 10. Adjusted estimates of the effect of glucagon-like peptide-1 receptor agonists (GLP-1RAs) on erectile dysfunction.**

| **Adjusting variable** | **OR (95% CI)** | ***P* value** | ***P*_pleiotropy_** | ***P*_heterogeneity_** |
| --- | --- | --- | --- | --- |
| **None** | **0.493 (0.430−0.565)** | **<0.001** | **0.899** | **1.000** |
| T2D | 0.503 (0.438-0.577) | <0.001 | 0.896 | 1.000 |
| Obesity | 0.517 (0.451-0.594) | <0.001 | 0.903 | 1.000 |
| Human ageing | 0.490 (0.427-0.563) | <0.001 | 0.887 | 1.000 |
| BMI | 0.498 (0.434-0.571) | <0.001 | 0.899 | 1.000 |
| Smoking | 0.493 (0.430-0.566) | <0.001 | 0.899 | 1.000 |
| Drinking | 0.486 (0.423-0.557) | <0.001 | 0.892 | 1.000 |
| CVD | 0.504 (0.439-0.578) | <0.001 | 0.922 | 1.000 |
| Hypertension | 0.504 (0.439-0.578) | <0.001 | 0.931 | 1.000 |
| Metabolic syndrome | 0.494 (0.430-0.566) | <0.001 | 0.899 | 1.000 |
| Hyperlipidemia | 0.490 (0.427-0.562) | <0.001 | 0.903 | 1.000 |
| Antihypertensives | 0.480 (0.418-0.553) | <0.001 | 0.887 | 1.000 |
| Immunosuppressants | 0.492 (0.429-0.564) | <0.001 | 0.903 | 1.000 |
| NSAIDs | 0.488 (0.426-0.561) | <0.001 | 0.912 | 1.000 |

OR: odds ratio, CI: confidence interval;

T2D: type 2 diabetes, BMI: body mass index, CVD: cardiovascular disease;

NSAIDs: nonsteroidal anti-inflammatory Drugs.





**Supplementary Figure 1: Methodology for the analysis of mediation ratios.**


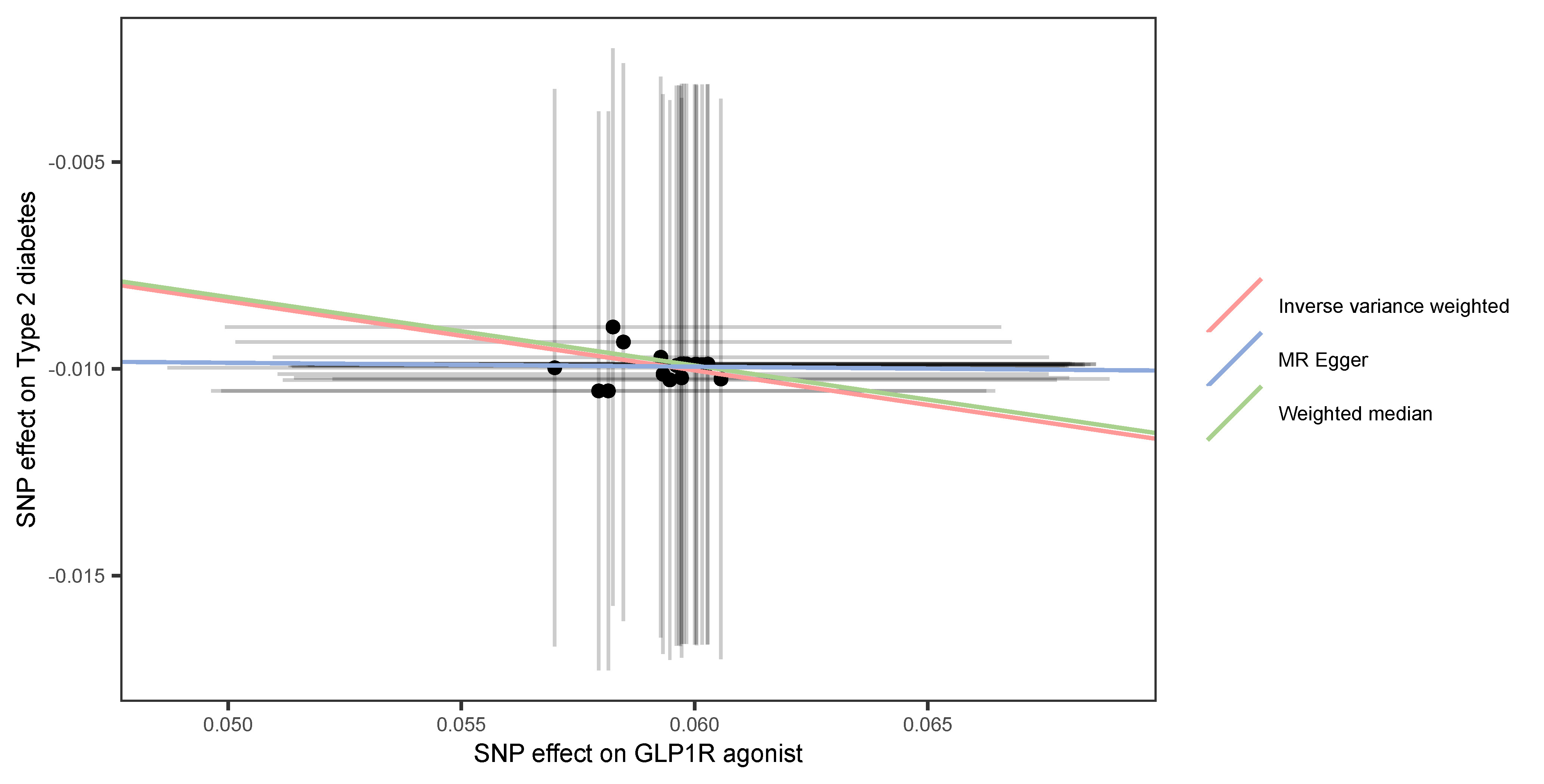


**Supplementary Figure 2.** Scatter plot of SNP effects on GLP1R agonist versus type 2 diabetes, with the slope of each line corresponding to the estimated MR effect per method. The data are expressed as raw β values with 95% CIs.

(SNP: single nucleotide polymorphism; GLP1R: glucagon-like peptide-1 receptor; MR: Mendelian randomization; CIs: confidence interval.)


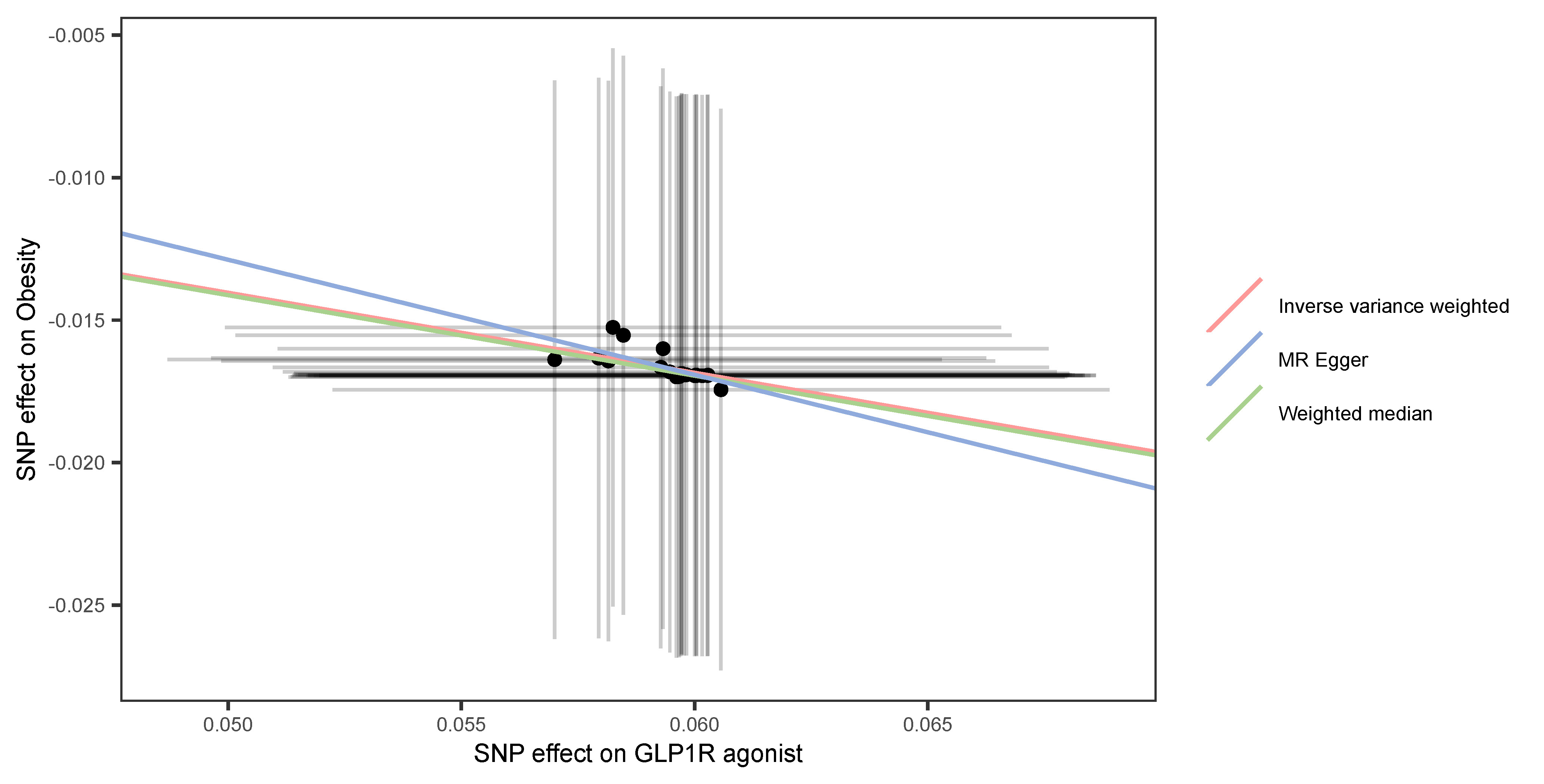


**Supplementary Figure 3.** Scatter plot of SNP effects on GLP1R agonist versus obesity, with the slope of each line corresponding to the estimated MR effect per method. The data are expressed as raw β values with 95% CIs.

(SNP: single nucleotide polymorphism; GLP1R: glucagon-like peptide-1 receptor; MR: Mendelian randomization; CIs: confidence interval.)


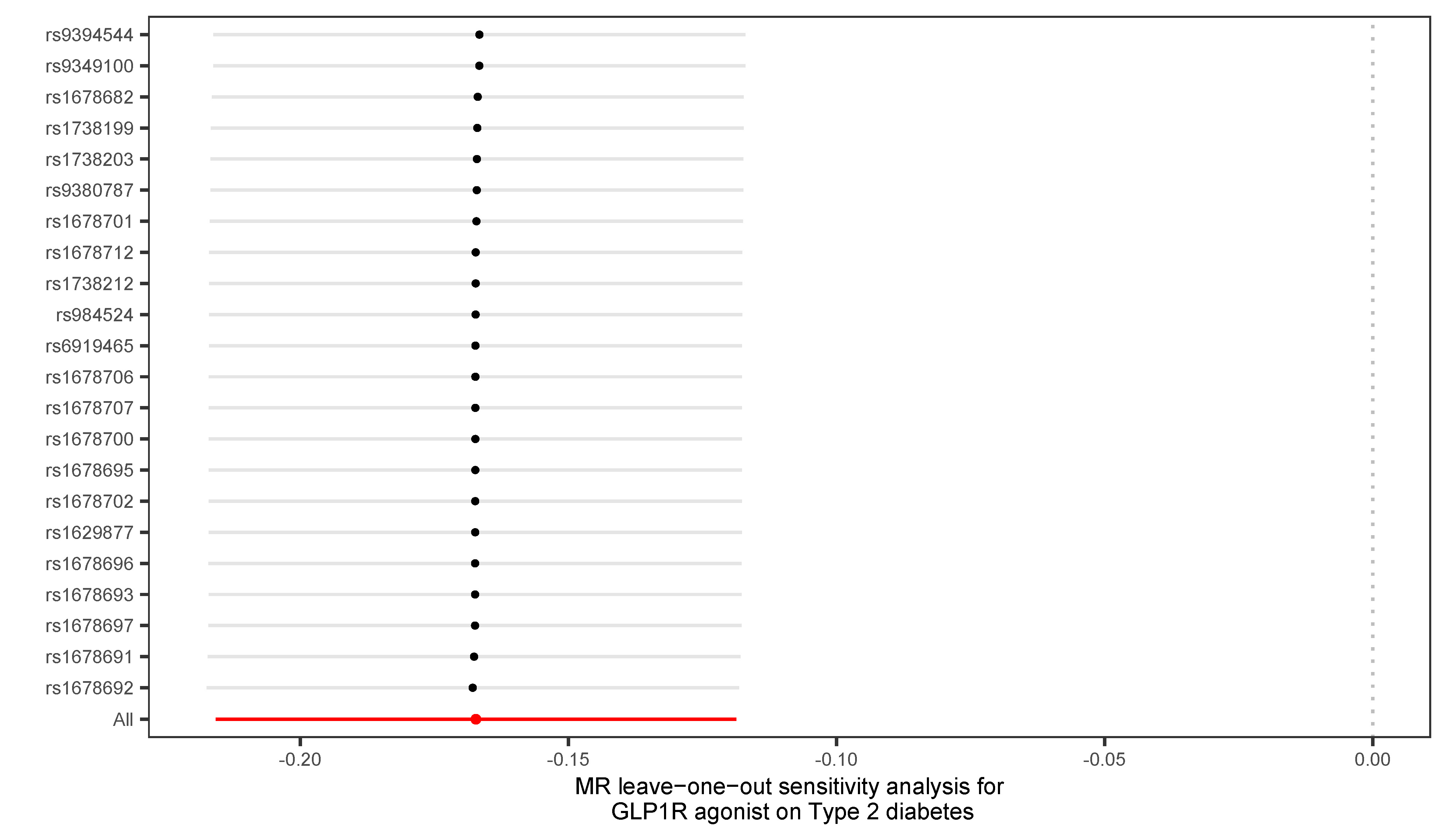


**Supplementary Figure 4.** The leave-one-out sensitivity analysis for GLP1R

agonist on type 2 diabetes.

(GLP1R: glucagon-like peptide-1 receptor; MR: Mendelian randomization.)


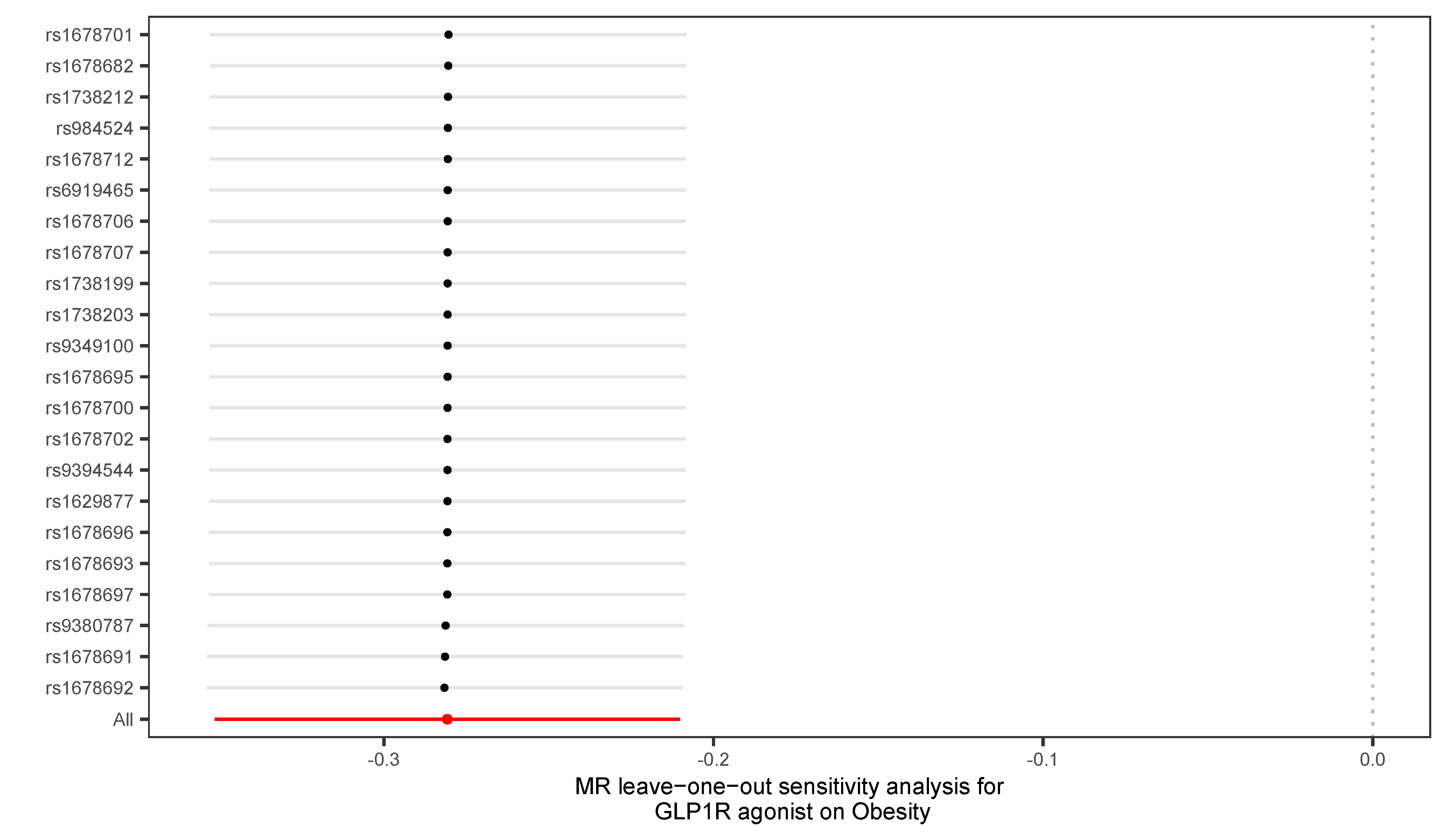


**Supplementary Figure 5.** The leave-one-out sensitivity analysis for GLP1R

agonist on obesity.

(GLP1R: glucagon-like peptide-1 receptor; MR: Mendelian randomization.)


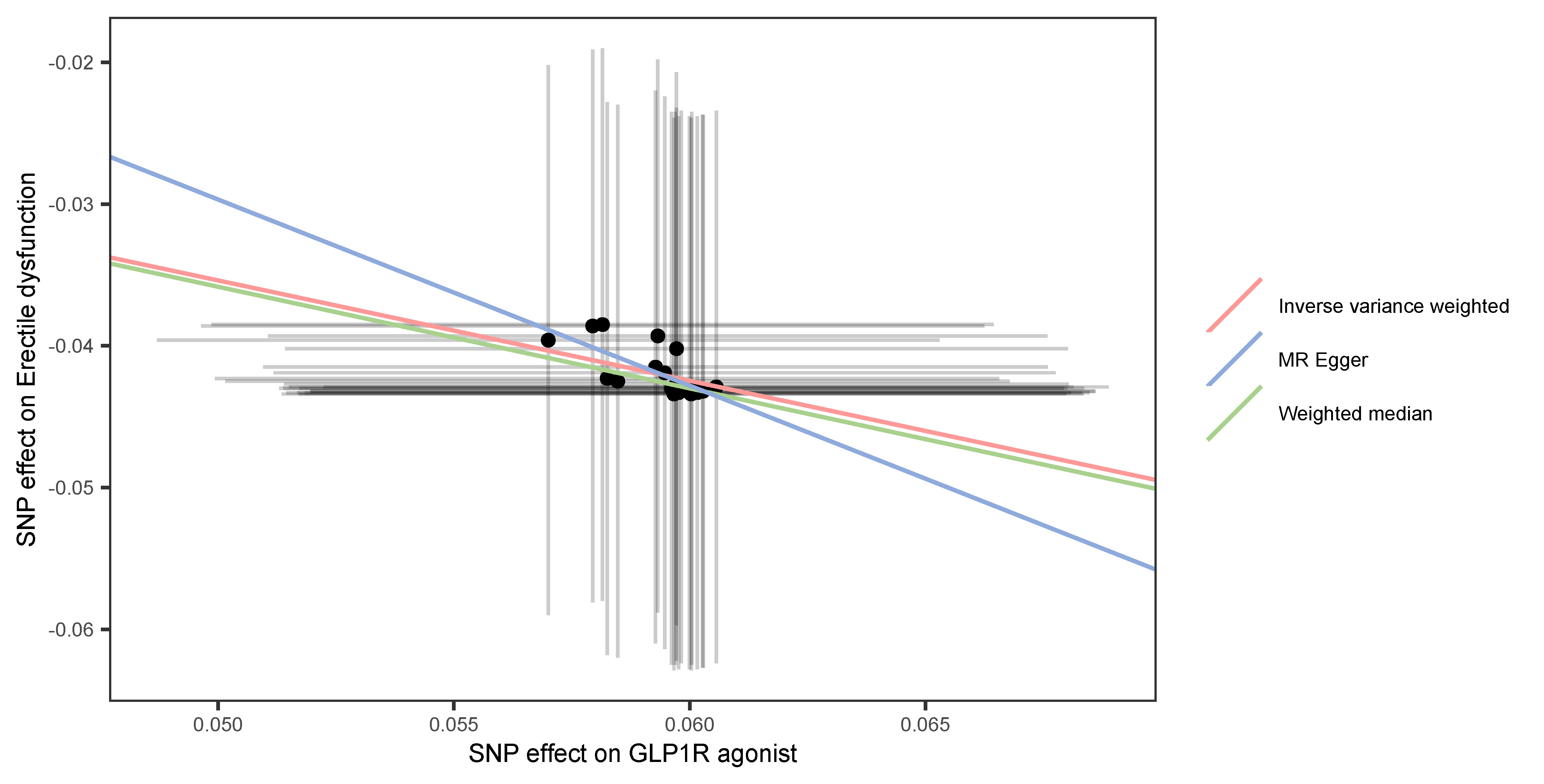


**Supplementary Figure 6.** Scatter plot of SNP effects on GLP1R agonist versus erectile dysfunction, with the slope of each line corresponding to the estimated MR effect per method. The data are expressed as raw β values with 95% CIs.

(SNP: single nucleotide polymorphism; GLP1R: glucagon-like peptide-1 receptor; MR: Mendelian randomization; CIs: confidence interval.)


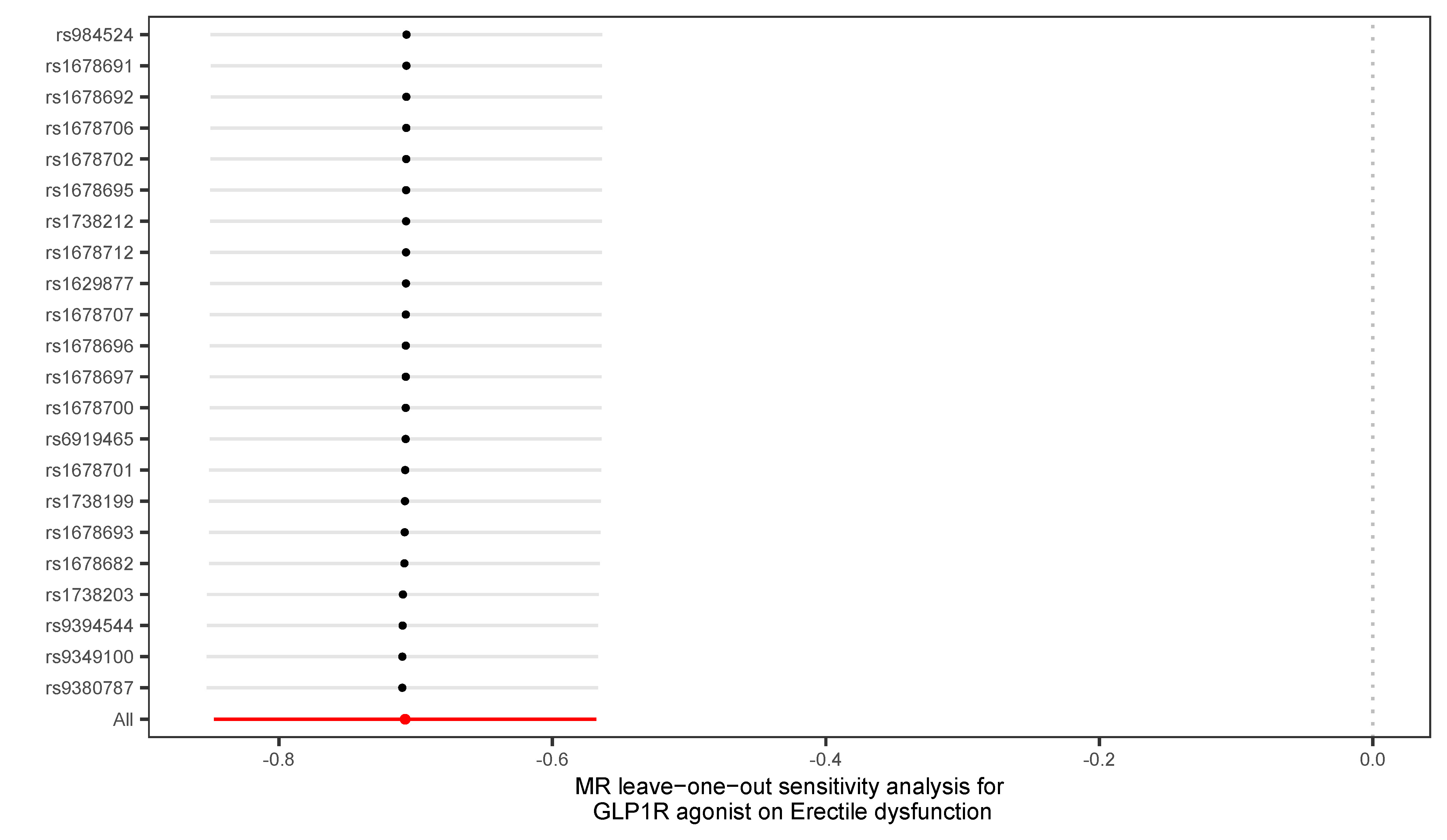


**Supplementary Figure 7.** The leave-one-out sensitivity analysis for GLP1R

agonist on erectile dysfunction.

(GLP1R: glucagon-like peptide-1 receptor; MR: Mendelian randomization.)
